# An integrative systematic review of nurses’ involvement in medication deprescription in long-term healthcare settings for older people

**DOI:** 10.1177/20420986241289205

**Published:** 2024-10-16

**Authors:** Mojtaba Vaismoradi, Abbas Mardani, Manuel Lillo Crespo, Patricia A. Logan, Natalia Sak-Dankosky

**Affiliations:** Faculty of Nursing and Health Sciences, Nord University, Universitetsalléen 11, Bodø 8049, Norway; Faculty of Science and Health, Charles Sturt University, Orange, NSW, Australia; Social Determinants of Health Research Center, Research Institute for Prevention of Non-Communicable Diseases, Qazvin University of Medical Sciences, Qazvin, Iran; Department of Nursing, Faculty of Health Sciences, University of Alicante, Alicante, Spain; Faculty of Science and Health, Charles Sturt University, Bathurst, NSW, Australia; Department of Clinical Nursing, Medical University of Warsaw, Warsaw, Poland

**Keywords:** deprescription, long-term care, medication management, nurse, patient safety

## Abstract

**Background:**

Deprescription of medications for older people in long-term care settings is crucial to enhance medication safety by reducing polypharmacy and minimizing related adverse events. Nurses as the member of the multidisciplinary healthcare team can support deprescription initiatives, but there is a gap in comprehensive knowledge about their roles.

**Objectives:**

To investigate the role and contribution of nurses in deprescribing medications within the multidisciplinary pharmaceutical care context of long-term healthcare for older people.

**Design:**

A systematic review utilizing an integrative approach was performed.

**Methods:**

Multiple databases were searched, including PubMed (covering MEDLINE), Scopus, CINAHL, ProQuest and Embase, focusing on studies published in English from 2014 to 2024. The preliminary search yielded 4872 studies, which were then refined to 32 qualitative and quantitative studies chosen for data analysis and narrative synthesis. Thematic comparisons and analysis led to the creation of meaningful categories integrating the studies’ findings to meet the review’s objective.

**Results:**

The review findings were classified into categories: ‘necessity and benefits of deprescribing’, ‘multidisciplinary collaboration for deprescribing’, ‘nurse role in deprescribing’, ‘identified challenges to deprescribing’, ‘involvement of older people and families in deprescribing’. They illustrated and exemplified various aspects of nurses’ roles and contributions in deprescription initiatives within the multidisciplinary pharmaceutical care team, such as support for reducing doses, discontinuing medications or transitioning to safer alternatives, as well as factors influencing this process.

**Conclusion:**

The main dimensions of nurses’ roles and contributions in deprescription initiatives encompass monitoring, communicating and educating. Challenges to nurses’ active participation in deprescribing, such as the need for increased knowledge, confidence and inclusion in team discussions, should be addressed through education, training and changing attitudes. These steps are essential for improving the safety of medication deprescribing in long-term care settings.

**Trial registration:**

The review was registered under PROSPERO ID: CRD42023486484, and can be accessed at crd.york.ac.uk/PROSPERO/display_record.php?RecordID=486484

## Plain language summary

Nurses’ role in stopping medications for older patients in long-term care within a team-based healthcare approach

**Why was the study done?** The number of medications older people take in long-term care should be reduced to enhance their safety. Nurses play a key role in this process, but not much is known about their roles and contributions. Therefore, this study aimed to explore nurses’ roles in stopping medications in the context of long-term care for older people.

**What did the researchers do?** A systematic review of international literature was conducted, searching multiple online databases for English-language studies published between 2014 and 2024. Out of 4872 studies, 32 were selected for detailed analysis and categorization.

**What did the researchers find?** Our findings highlighted the importance of medication discontinuation and its benefits, and the collaborative efforts of nurses within the healthcare team. Obstacles faced by nurses and healthcare staff, and how older people and their families could be actively involved in the decision-making process were also identified.

**What do the findings mean?** We concluded that nurses should have a more active role in stopping medications by monitoring, communicating, and educating. However, they need training and support, boosting their confidence, and being included in the process to improve medication safety in long-term care settings for older people.

## Introduction

It has been estimated that the number of individuals older than 60 years by the year 2050 will double, reaching 2.1 billion.^1^ The rapid ageing of the population and the limited capacity of the current healthcare system to care for older people have led to the development of the long-term care system characterized by the rapid growth of the residential care sector.^2^ Long-term care includes services for individuals needing assistance with daily activities and often combines personal care with basic medical support, such as wound care, pain management, medication, health monitoring and rehabilitation or palliative care.^3^ The continuity of care is the main underlying concept of long-term care for the prevention of hospital readmissions and related consequences for both older people and the healthcare organization.^4^

Having chronic diseases and multiple medical conditions among older people requires the use of various medications. Therefore, interventions that best support medication continuity for older people at long-term care settings are particularly effective in improving the quality and safety of care.^5^ Besides functional deficits and increased comorbidities, issues with medication management are significant risk factors for hospitalization among these older people.^6^ It has been shown that up to 91%, 74% and 65% of older people in long-term care facilities are prescribed more than 5, 9 and 10 medications, respectively.^7^ Polypharmacy poses a significant threat to older people, as it heightens the risk of falls, cognitive decline and mortality.^8,9^ Also, excessive polypharmacy (OR = 1.66, *p* = 0.007) has been found to significantly increase the odds of readmission^10^ and the risk of medication-related readmissions.^11^ In addition, 53% of these older people have at least one potentially inappropriate medication (PIM) with a greater number of medications significantly associated with the likelihood of PIM^12^ and older people’s frailty.^13^

The use of warfarin, Nonsteroidal Anti-Inflammatory Drugs (NSAIDs), pantoprazole and vinpocetine in older people is linked to hospitalizations, with controversial results for long-term use of aspirin, statins, trimetazidine, digoxin and β-blockers.^14^ Antipsychotic use is 17.2% and 6.6% among those with Alzheimer’s and those without it, respectively, predicted by higher medication burden (OR = 1.04, *p* = 0.02) and behavioural symptoms (OR = 5.26, *p* = 0.002), and is associated with less improvement in daily activities (β = −0.70, *p* < 0.001).^15^ Therefore, optimizing medication use can reduce drug-related problems, morbidity and mortality from polypharmacy.^8^

Deprescribing, which involves stopping or reducing medications, is advocated as beneficial practice aiming at improving the longevity and well-being of older people.^9^ It has been recognized as a medication management strategy for reducing polypharmacy and eliminating PIM.^16^ Integrating deprescribing into routine care significantly increases medications’ discontinuation without raising the rates of emergency department visits or hospital admissions.^17^ These interventions directed by medication review can decrease all-cause mortality by 26% (OR = 0.74, 95% confidence interval (CI): 0.65–0.84), number of falls by 24% (OR = 0.76, 95% CI: 0.62–0.93) and even can result in modest reductions in mortality rates in nursing homes.^18^

International literature emphasizes the critical role of nurses in identifying clinically significant drug-related issues and errors that can harm older people. Nurses can contribute to reporting these issues to physicians and pharmacists, who then make further decisions about medication prescription and deprescription.^19,20^ Current literature provides substantial insight into the roles of pharmacists and physicians in deprescription practices. However, there is a notable gap regarding the roles of nurses in this area. Investigating the specific roles and contributions of nurses in deprescription interventions carries significant implications for clinical practice in terms of enhancing interprofessional collaboration, optimizing patient care outcomes and informing evidence-based practice guidelines. Therefore, this review aimed to investigate the role and contributions of nurses in deprescribing medications within the multidisciplinary pharmaceutical care context of long-term healthcare for older people. Accordingly, the review question was: ‘What are nurses’ roles and contributions to deprescribing medications within the pharmaceutical care context for older people in long-term care?’

## Methods

### Design

A systematic review with an integrative approach was conducted. The integrative approach was employed to synthesize existing knowledge comprehensively, combining diverse sources of evidence, including quantitative and qualitative data. The integrative method offers a more thorough understanding of the study phenomenon by capturing different dimensions of the topic. Qualitative studies offer in-depth insights into experiences and perspectives from healthcare professionals’ perspectives, while quantitative studies provide measurable evidence, and their integration informs more robust conclusions and recommendations.^21^

The review adhered to the Preferred Reporting Items for Systematic Reviews and Meta-Analyses (PRISMA) guidelines for its development and reporting (Supplemental File 1). It followed a five-step process: identifying the research problem, conducting an extensive literature search, appraising the quality of the data, analyzing and synthesizing the data and presenting the findings.

### Protocol and registration

A multinational team of researchers with expertise in patient safety, medication management and systematic review methodology was assembled to collaboratively develop the review protocol. To enhance transparency, ensure the integrity of the review process and reduce the risk of publication bias, the review protocol, including the objectives, methods and analysis plan, was registered under the PROSPERO ID: CRD42023486484, accessible via the following link: crd.york.ac.uk/PROSPERO/display_record.php?RecordID=486484.

The objectives and approach of the review were formulated using the PICo framework as follows:

- *P (Population)*: Clinical nurses, including practical nurses, registered nurses, nurse practitioners or licensed practical nurses, providing care within the multidisciplinary healthcare team consisting of physician, pharmacist, older people, caregivers and families.
- *I (Intervention)*: Initiatives for deprescribing medications, including directives to discontinue the use of both prescription and non-prescription medications (over-the-counter (OTC) and pro re nata (PRN)) to reduce unnecessary polypharmacy and avoid medications’ side effects and adverse drug reactions with nurses being involved in developing and implementing deprescribing strategies, managing processes and improving overall medication management.
- *C (Context)*: Long-term care providing extended care for older people with chronic illnesses, disabilities or conditions requiring ongoing assistance. Examples include nursing homes, skilled nursing facilities, assisted living facilities, hospice care, rehabilitation centres, home healthcare services, adult day care centres and residential care facilities.
- *O (Outcome)*: Various outcomes related to medication safety such as reduced medication use and medication errors, and improved adherence to medication regimens, with a positive impact on the well-being of older people and family/informal caregivers.

### Literature search

This review was motivated by the lack of comprehensive reviews that integrate an understanding of the study phenomenon. Our extensive literature search encompassed multiple health-related databases, including PubMed (including MEDLINE), Scopus, CINAHL, ProQuest and Embase. The search spanned the last decade, from 1 January 2014 to 30 April 2024, to ensure that the findings reflected the most current research, practices and trends relevant to the study phenomenon.

Initially, keywords relevant to the review topic were identified through Google Scholar, leveraging personal research experiences. Search strings were developed by translating Medical Subject Headings and thesaurus terms into compatible terms for the selected databases. Boolean logic and truncation were applied in search queries, using operators like AND/OR. A librarian was consulted to validate and enhance the search accuracy.

Multiple variations of key terms related to nurse, medication management, long-term care, older people and deprescribing were utilized (Supplemental File 2). Furthermore, references from retrieved articles and current review papers were examined to broaden search coverage. Grey literature sources, including contemporary reports on medication management by the multidisciplinary pharmaceutical care team, were identified through a Google search.

### Inclusion and exclusion of studies

All original research studies utilizing qualitative, quantitative and mixed-methods designs were assessed for inclusion based on rigorous scientific criteria. The selection focused on studies that:

- Investigated medication management;
- Took place in long-term care settings;
- Involved older adults (⩾60 years) as the primary age group;
- Involved nurses by assigning them specific roles and contributions in the deprescribing process within the multidisciplinary pharmaceutical care team;
- Were published in peer-reviewed scientific journals in English.

Exclusions encompassed:

- Commentaries, letters, reviews, conference proceedings and books;
- Studies involving age groups other than older adults;
- Research conducted in acute and ambulatory healthcare settings such as emergency departments and hospitals;
- Studies published before 2014.

Search results were uploaded to the Rayyan online platform for systematic reviews for screening. Two review authors (M.V. and N.S.-D.) independently conducted systematic screening and selection of studies, adhering to eligibility criteria applied to titles, abstracts and full texts. Initial screening involved assessing titles and abstracts against predefined criteria before proceeding to full-text evaluation. Consensus on study selection and inclusion for reporting was achieved through collaborative discussions and shared findings. Detailed tables summarizing study details were created, and the selection process was systematically documented to facilitate transparency and discussion of reasons for inclusion and exclusion. Following initial screening, review authors discussed findings and next steps via MS Teams, resolving discrepancies through consensus. In cases of disagreement, the perspective of the other review author was sought to ensure thorough consideration and final decision-making.

### Quality appraisal

It encompassed a thorough assessment of credibility, pertinence and findings conveyed in the selected studies by three review authors (M.V., A.M., P.A.L.). The Mixed Methods Appraisal Tool (MMAT), version 2018 was used for appraising studies with diverse methodologies due to its comprehensive and structured approach. It provides clear criteria for evaluating qualitative, quantitative and mixed-methods studies, ensuring rigorous assessment of study quality across various research contexts. MMAT’s user-friendly design and evidence-based development facilitate a consistent and reliable evaluation of methodological rigor.^22^

Additionally, for assessing the risk of bias in randomized clinical trials, the RoB 2 assessment tools (2024) were used.^23^ The robvis tool (Risk of Bias Visualisation) was employed to depict the assessment report. The review authors (M.V., A.M., P.A.L.) independently evaluated the studies and provided comprehensive explanations of their perspectives on the studies’ methodological qualities. A joint determination regarding whether to include or exclude studies in the research synthesis was made. This decision-making process considered the importance, methodological quality and potential bias, ensuring a rigorous and well-informed selection process.

### Research synthesis

Substantial variations in research methodologies among the included studies ranging from clinical trials and quasi-experimental studies to cross-sectional studies, each differing in objectives, data gathering tools and measurement outcomes were revealed. Additionally, the inclusion of qualitative and mixed-methods studies prevented the possibility of conducting a meta-analysis. Therefore, the review findings were presented through a descriptive and narrative approach, covering both statistical and non-statistical details of the qualitative and quantitative studies to offer a comprehensive understanding of the review topic. Data synthesis from the selected studies was facilitated using an extraction table, which organized, summarized and compared general and specific characteristics of studies. Collaborative teamwork involved integrating study findings through thematic comparisons and analysis, resulting in the development of meaningful categories that synthesized findings to achieve the review’s objectives. Key codes and concepts were initially identified through reading and coding of extracted data from the included studies, which were classified based on their similarities and differences to create categories. Continuous discussions among the review team members led to refining and validating these categories to capture essential aspects within data. Attention was consistently given to the context within each study, ensuring that the synthesis of findings remained grounded in the original data and integrated diverse perspectives and insights from the included studies.

### Ethical considerations

Ethical approval was unnecessary for this study as it did not involve human samples. However, the review authors adhered to maintaining transparency and honesty in reporting, minimizing bias and upholding proper citation practices to respect intellectual property rights.

## Results

### Search outcome and study selection

**Figure 1.**
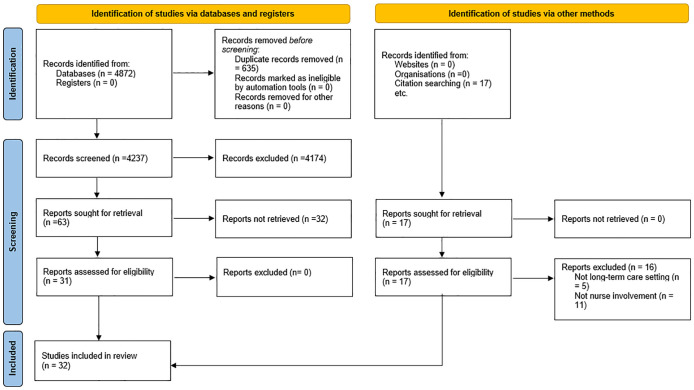
The search results are based on the PRISMA. PRISMA, Preferred Reporting Items for Systematic Reviews and Meta-Analyses.

The initial database search resulted in 4872 studies (Supplemental File 3). After removing duplicates and excluding studies based on titles and abstracts that did not meet the inclusion criteria, 63 studies remained. A detailed full-text review further narrowed this down to 32 articles. The primary reasons for exclusion were that the studies did not involve nurses in deprescription interventions or focused on acute and ambulatory healthcare settings like hospitals. Figure 1 visually illustrates the search process according to PRISMA.

### Methodological quality appraisal

The randomized clinical trials^24–30^ demonstrated a proper causal relationship between variables, as well as rigorous methods for sampling, group assignment, follow-up procedures and outcome measurement. These studies generally exhibited a low risk of bias related to confounding factors, participant selection, intervention implementation, data collection, measurement and result reporting. Regarding the risk of bias assessment, some concerns were noted regarding participant selection and sample representativeness, assessors’ blinding, randomization process and missing outcome data.

The quasi-experimental studies^31–35^ provided clear and consistent measurements of intervention and outcome variables, detailed implementation and contextual factors, ensuring transparency and reproducibility. However, some concerns about group similarities and their treatment were noted.

For the qualitative studies,^36–47^ the appropriateness of the research design to answer the research question, transparency in data collection and analysis, sufficiency of data interpretation and coherence between data sources, collection, analysis and interpretation were all well-established.

In the mixed-methods studies,^48–51^ key aspects such as an adequate rationale for using such a design, integration of different components to answer the research question, and coherence between qualitative and quantitative components were well-established. Additionally, divergences and inconsistencies between quantitative and qualitative results were addressed, with each component adhered to the quality criteria of its respective methodological tradition.

In the cross-sectional studies,^52–55^ the relevance of the sampling strategy to address the research question, representativeness of the sample, appropriate risk measurement of nonresponses and appropriateness of statistical analysis were all identified as key aspects. All 32 studies met the criteria for adequate methodological quality and were subsequently included in the data analysis and research synthesis (Supplemental File 4).

### General characteristics of included studies

All included studies were published in English and spanned the past 10 years from 2014 to 2024. Table 1 presents a summary of the selected studies, detailing sample and setting, nurse qualification and role in deprescription initiatives and implications for patient safety.

**Table 1.** The general characteristics of the selected studies.

Type of long-term care setting	Authors, year/country	Methodology	Sample	Nurse classification	Nurse role in deprescription initiative within the study	Impact on patient safety
Nursing home	Abrahamson et al., 2021/United States^36^	Comparative case study	62 Staff members and staff at 15 nursing homes and 14 assisted living facilities	Registered nurses/nursing assistant	Storytelling, aromatherapy, exercise interventions	Monitoring processes and outcomes of care
	Ailabouni, et al., 2017/Canada^52^	Cross-sectional survey	307 Registered nurses	Registered nurse	Nurses’ views on deprescribing	Increased awareness regarding polypharmacy and potential deprescribing benefits
	Azermai et al., 2014/Belgium^48^	Mixed-method design	4 Nursing homes: 226 nurses and nurse assistants	Responsible nurses (primary caregivers)	Willingness and barriers to undertake antipsychotic discontinuation	Need for a complex multidisciplinary intervention
	Brodaty et al., 2018/Australia^31^	Repeated-measures, longitudinal, single-arm	139 Residents from 23 nursing homes	Residential care nurses	Nonpharmacological prevention and management of symptoms	Withdrawal was not accompanied by drug substitution or a significant increase in PRN antipsychotic or benzodiazepine administration
	Cateau et al., 2021/Switzerland^25^	Clinical trial	58 Older people at 7 nursing homes	General nurse	Individualized treatment modification plan in collaboration with nurses	Identification of adverse events that required the reintroduction of withdrawn treatments
	Evrard et al., 2020/Belgium^26^	Cluster controlled trial	797 Older people in 54 nursing homes	General nurse	Recording administrative, and clinical data	Appropriateness of use and identification of factors associated with benzodiazepines and deprescribing
	Gedde et al., 2021/Norway^27^	Four-month multicentre, multicomponent, cluster-randomized, single-blinded controlled trial	428 Older people at 33 Norwegian nursing homes including 67 nursing home wards	General nurse	Cooperation in medication review	Medication reviews using collegial mentoring and systematic clinical evaluation
	Gulla et al., 2018/Norway^28^	Multicentre, cluster-randomized, controlled trial	765 Older people from 72 units in 32 Norwegian nursing homes	Not mentioned	Multidisciplinary medication review, and organization of deprescription activities	Collegial mentoring
	Hølmkjær et al., 2022/Denmark^50^	A stepwise process	5 GPs, 2 experts, 3 older people/relative dyad, 3 nursing home staff groups	General nurse	Medication and their experience with deprescribing of antidepressants and other psychotropic medication	Thorough development process, in future expects to achieve increased adherence to the intervention
	Kua et al., 2019/Singapore^39^	Qualitative	4 Doctors, 4 pharmacists and 9 nurses	Staff nurses or enrolled nurses	Monitoring side effects and efficacy	Challenging nature of deprescribing
	Kua et al., 2021/United States^29^	Pragmatic multicentre stepped-wedge cluster randomized controlled trial	295 Older people from 4 nursing homes	General nurse	Discussion with nurses on the feasibility of deprescribing for each resident, with an option to discuss with cognitive-intact residents (or family members of cognitively impaired residents); communication through nurse to physician for reviewing and deprescribing decisions	Association between multidisciplinary medication review-directed deprescribing and reductions in mortality and number of hospitalized residents
	McConeghy et al., 2022/United States^32^	Retrospective study	3247 Older people at 64 nursing homes	General nurse	Patient assessment to identify and address clinical issues associated with holding medications	Organization-wide initiative for deprescribing
	Niznik et al., 2022/United States^33^	Quasi-experimental	5312 Older people	Nurse practitioners	Deprescribing of oral bisphosphonates, defined as a 90-day gap in medication supply	Nurse practitioners may be more attuned to an individual resident’s prognosis and may have more opportunity to scrutinize the continued appropriateness of medications on an ongoing basis
	Pruskowski et al., 2019/United States^55^	Longitudinal deprescribing-focused experience	44 Older people	Not mentioned	Documenting proper medication use, making recommendations to prescribers for optimal use, evaluating medication distribution by the nurses	Practice empathy, critical thinking and communication
	Simmons et al., 2018/United States^41^	Qualitative	29 Staff in three community nursing homes	Licensed nurse, registered nurse, social worker, facility administrator, nurse practitioner, director-of-nursing, certified nursing assistant, assistant director-of-nursing and mental health intern	Benefits of and barriers to reducing inappropriate use of medications	Multiple barriers identified for implementing reduction strategies in routine clinical practice
Long-term care facility	Balsom et al., 2020/Canada^24^	Randomized controlled trial	45 Older people (*n* = 22 intervention, *n* = 23 control)	Ward nurse	Taking a medication-focused clinical history	Pharmacist-led deprescribing intervention
	Baqir et al., 2017/United Kingdom^53^	Retrospective analysis	422 Care home residents in 20 care homes	Care home nurse	Medication optimization review by a team	Simplified and optimized medication regimen
	Chenoweth et al., 2018/Australia^38^	Qualitative	22 Nurses as champions at long-term care home	Registered nurse	Practical strategies for problem solving issues with individual resident behaviours	Person-centred approaches
	Palagy et al., 2016^40^	Qualitative	8 GPs, pharmacists, 19 nursing staff, 25 residents and 16 relatives	Registered nurse	Observation for identifying any medication-related issues	Need for education about identifying medications’ effects and side effects
	Perri et al., 2022/Canada^51^	Mixed methods design	55 Older people at two units of long-term care home, 2 physicians	General nurse	Medication review in the team	Flagging PIMs, integration in the electronic medical record
	Pruskowski and Handler, 2017/United States^54^	Quality improvement project	47 Older people at nursing facility	Nurse practitioner	Recommendations regarding the discontinuation of medications	Interprofessional collaboration, and the operational process
	Turner et al., 2016/Australia^44^	Qualitative	11 Residents, 19 physicians, 12 nurses and 14 pharmacists	General nurse	Review of medical conditions aligned to medications	Similarities and differences within the range of factors prioritized by residents and health professionals
	Warmoth et al., 2023/United Kingdom^47^	Qualitative	23 Care home staff, 8 older people, 4 family members and 1 general practitioner at 15 care homes	General nurse	Being consulted by the physician to make their decisions	Need for training, tools, support and opportunities to care home staff
	Westbury et al., 2018/Australia^35^	Multi-strategic, interdisciplinary program	2157 Older people at 150 aged care facilities	General nurse	Practice leaders working with various disciplines to ensure that guideline implementation and recommendations saturate an organization	Over-reliance on psychotropic medications for managing mental and psychological symptoms
Home care	Birt et al., 2022/United Kingdom^37^	Qualitative approach	6 Pharmacists, 6 physicians and 7 care home staff	General nurse	Independent prescribing	Clinical competence and professional willingness
	Drewelow et al., 2022/Germany^49^	Mixed-methods design	Two older people and their relatives	General nurse	Expert discussions	Structured family conference with a medication check and geriatric assessment
	Sheppard et al., 2020/United Kingdom^30^	Randomized, unblinded, noninferiority trial	Older people, intervention, (*n* = 282) or usual care (control, *n* = 287)	General nurse	Data collection in clinics at baseline, 4-week safety visit (for the intervention group only) and 12-week follow-up	A strategy of medication reduction, compared with usual care, was noninferior about systolic blood pressure control at 12 weeks
	Sun et al., 2019/Canada^42^	Exploratory qualitative descriptive research, thematic analysis	11 Home care nurses	Home care nurses	Learning and educational needs, barriers and enablers, exploration of non-pharmacological alternatives	Educational programmes to support awareness and understanding of deprescribing
	Sun et al., 2021/Canada^34^	Evaluation research study	45 Homecare nurses	Homecare nurse	Deprescribing at home	Barriers impacting the effectiveness of deprescribing education
	Tjia et al., 2019/United States^43^	Qualitative content analysis	10 Home and inpatient hospice nurses, drawn from 3 hospice agencies and their referring hospital systems	Hospice nurses, inpatient hospice nurse and medical home nurse coordinator	Medication management and deprescribing	Nurses’ willingness to discuss deprescribing with family caregivers and prescribers when conversations are framed around medication harms and their impact on quality of life
	Wang et al., 2023/United States^45^	Qualitative content analysis	14 Home health care older people, 15 practitioners (including 9 primary care physicians, 4 pharmacists, 1 hospitalist and 1 nurse practitioner), and 15 home health care nurses	Nurse practitioner, home health care nurse	Deprescribing at home	Patient-centred deprescribing
	Wang et al., 2024/United States^46^	Qualitative individual interviews	9 Older people, 11 nurses, 5 primary care physicians, 3 pharmacists, 1 hospitalist and 1 post-acute nurse practitioner from 9 centres	Nurse practitioners and registered nurses	Challenges in deprescribing medications for post-acute home care patients	Limited roles of nurses and pharmacists in care team collaboration and discussion about post-acute deprescribing

GP, general physician; PRN, *pro re nata*.

### Deprescription interventions and the involvement of nurses

**Figure 2.**
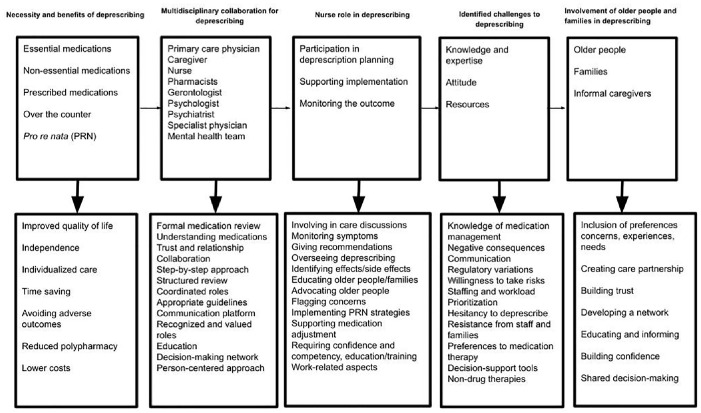
A schematic summary of the review results.

The studies’ statistical and nonstatistical results in relation to the review’s aim have been presented in Table 2. The review findings were classified into categories of ‘necessity and benefits of deprescribing’, ‘multidisciplinary collaboration for deprescribing’, ‘nurse role in deprescribing’, ‘identified challenges to deprescribing’, ‘involvement of older people and families in deprescribing’. These categories integrated the various aspects described and exemplified in the selected studies with respect to the nurses’ involvement and roles in deprescription initiatives within the multidisciplinary pharmaceutical team such as support for reducing doses, discontinuing medications or transitioning to safer alternatives, as well as factors influencing this process. Figure 2 provides a schematic representation of the review results.

**Table 2.** A summary of the findings from the selected studies in relation to the review’s aim.

Research design	Author, year	Outcome of the intervention for deprescribing
Quantitative	Ailabouni et al., 2017^52^	The top suggested medications for deprescribing were preventive medications, such as aspirin and statins (19%), antihypertensive agents (17%) and antipsychotic agents (15%); improving medication adherence (44%) and quality of life (50.5%).
	Balsom et al., 2020^24^	85.1% of deprescription cases were successful; on average, 2.68 fewer medications than the control group at 3 months (*p* < 0.02; 95% CI: −4.284, −1.071) and 2.88 fewer at 6 months (*p* = 0.02, 95% CI: −4.543, −1.112) were taken.
	Baqir et al., 2017^53^	704 Medications were stopped; 298 (70.6%) had at least one medication discontinued, 19.5% of the originally prescribed medications (*n* = 3602). The most commonly deprescribed medication groups were laxatives (14.5%), skin products (8.4%), bone protection drugs (7%), acid-regulating medications (5.4%), antidepressants (4.7%), antihypertensives (4.3%) and lipid-regulating medications (4.3%). Older people were closely monitored post-deprescribing, with adverse events documented and follow-up assessments conducted 1 month later. Only seven adverse events (0.99%) were reported. The discontinued medications resulted in annualized savings of £65,471.
	Brodaty et al., 2018^31^	Regular antipsychotics were deprescribed for 69/93 (74.2%) for 11.5 months (range: 9.4–14.4 months); regular antipsychotic medications’ stopped for 94.7% of participants, with complete cessation after 27 days (range: 0–78 days); no significant increase in PRN antipsychotic prescribing (*b* = 0.3 mg/month, *p* = 0.33, 97.5% CI: 1.0–0.4, t = 0.98, df = 474) or administration (*b* = 0.9 mg/month, *p* = 0.31, 97.5% CI: 3.0–1.1, t = 1.03, df = 110) following the deprescribing of regular antipsychotics; At the 3-, 6- and 12-month follow-ups, respectively, 93.5%, 87.3% and 90.3% of participants received none or less than half of their original dose of regular antipsychotics.
	Cateau et al., 2021^25^	169 Treatment modifications were proposed; 82 (49%) were implemented, 67 were sustained at follow-up. Most propositions concerned pain medications (20 proposed, 10 accepted, 9 sustained), benzodiazepines (16 proposed, 6 accepted, 3 sustained), PPIs (13 proposed, 6 accepted, 5 sustained) and blood pressure drugs (11 proposed, 3 accepted, 3 sustained). A significant reduction in PIMs dose, with a 24% reduction overall (IRR = 0.763, 95% CI: 0.594–0.979) and a 28% reduction in chronic PIMs (IRR = 0.716, 95% CI: 0.546–0.938) was observed.
	Evrard et al., 2020^26^	Benzodiazepine use decreased from 52.3% (237/453) at baseline to 47.2% (214/453) at the end of the study, indicating an absolute decrease of 5.1%; Of benzodiazepine users, 32.9% (78/237) underwent deprescribing; 47.4% (37/78) completely ceased benzodiazepine prescriptions.
	Gedde et al., 2021^27^	Patients regularly prescribed three or more psychotropic drugs at baseline (*n* = 31) experienced a greater mean reduction compared to the control group (*n* = 36, df = 67, *p* < 0.001). Hypnotics or sedatives (df = 426, *p* = 0.011) and antidepressant drugs (df = 426, *p* = 0.041) use were reduced compared to the control group.
	Gulla et al., 2018^28^	Between baseline and month 4, antihypertensives were deprescribed significantly more in the intervention group (32%) compared to the control group (10%); IRR = 0.8, 95% CI: 0.7–0.9. Systolic blood pressure increased from 128 ± 19.5 mmHg to 143 ± 25.5 mmHg when antihypertensives were reduced but returned to baseline (mean 134 mmHg) by month 9. Hospitalizations were higher in the control group at both month 4 (*p* = 0.031) and month 9 (*p* = 0.041).
	Kua et al., 2021^29^	Reduced mortality (2.9%, HR: 0.16, 95% CI: 0.07–0.41; *p* < 0.001) and fewer hospitalized residents (7.3%, HR: 0.16, 95% CI: 0.10–0.26; *p* < 0.001), along with decreased regular pill burden (0.67, *p* = 0.001), PRN pill burden in those under 80 years old (*n* = 0.47, *p* = 0.014), and an estimated daily cost saving of US$11.42 (SG$15.65) for regular and PRN medications.
	McConeghy et al., 2022^32^	54% (2897/5297) of medications were permanently discontinued. Probiotics had the highest discontinuation rate at 73%, followed by histamine-2 receptor antagonists (66%), antihistamines (64%) and statins (45%); discontinuation rates by medication class ranged 45.5%–72.7%: statins (45.5%), PPIs (57.7%) and multivitamins (52.6%). Overall, 98% of fully stopped medications were discontinued.
	Niznik et al., 2022^33^	The 180 and 270 days cumulative incidences of deprescribing bisphosphonates were 14.8% and 20.4%, respectively.
	Perri et al., 2022^51^	A mean reduction of 1.6 medications per person with an IQR of 1.0 was achieved, while the standard method resulted in a mean reduction of 0.3 medications per person, also with an IQR of 1.0 (*p* = 0.02).
	Pruskowski and Handler, 2017^54^	The clinical pharmacist made 39 recommendations for 23 older people, averaging 0.82 recommendations per resident (range: 0–5). Only 10 (26%) were accepted, 1 (3%) was modified, 3 (7%) were rejected and 25 (64%) received no response within 120 days. Of the 10 accepted recommendations, 60% were supplements, 10% were antihyperglycemic, 10% were cardiovascular and 20% were gastrointestinal medications.
	Pruskowski et al., 2019^55^	Out of 44 residents, 69 recommendations were made, with 60 for deprescribing and 9 for other geriatric considerations. Over half of the deprescribing recommendations were for supplements, while approximately 25% were for cardiovascular medications and 13% for gastrointestinal medications. The primary team accepted 71% of the recommendations, with only 4% rejected. The remaining 25% received no response within the 120-day period.
	Sheppard et al., 2020^30^	At 12 weeks, 187 participants (66.3%) maintained medication reduction. The intervention group had a mean increase in systolic blood pressure of 3.4 mmHg (95% CI: 1.1–5.8 mmHg) compared to the control group. Serious adverse events were reported by 12 participants (4.3%) in the intervention group and 7 participants (2.4%) in the control group (adjusted RR, 1.72 (95% CI: 0.7–4.3)). At 12-week follow-up, the medication reduction group was taking 0.6 fewer antihypertensive medications than the usual care group. Additionally, 38.1% (95% CI: 32.2%–44.2%) of this group had no increase in systolic blood pressure.
	Westbury et al., 2018^35^	During the 6-month intervention, 40% of older people had antipsychotics and benzodiazepines reduced (15%) or ceased (24%). Antipsychotic prescriptions declined by 13% (from 21.6% to 18.9%) and benzodiazepine prescriptions by 21% (from 22.2% to 17.6%). Mean chlorpromazine dose decreased from 22.9 to 20.2 mg per resident/day and mean diazepam dose from 1.4 to 1.1 mg per person/day. No substitution with sedating antidepressants or other psychotropic agents. PRN antipsychotic prescriptions declined by 13% (*p* = 0.004) and benzodiazepines by 8% (*p* = 0.020).
Qualitative	Abrahamson et al., 2021^36^	Barriers to deprescription practice from healthcare staff perspectives.
	Birt et al., 2022^37^	Requirements for medication deprescription.
	Chenoweth et al., 2018^38^	Nurses’ needs and empowering them to implement deprescription strategies.
	Hølmkjær et al., 2022^50^	Nurse-physician professional relationship influencing collaboration in deprescription initiatives.
	Kua et al., 2019^39^	Facilitators and obstacles from the perspectives of doctors, pharmacists and nurses in deprescribing.
	Palagy et al., 2016^40^	Factors influencing the use and deprescription of medications from various healthcare providers, older people and relatives.
	Simmons et al., 2018^41^	Benefits of and barriers to deprescribing from nurses’ perspectives.
	Sun et al., 2019^42^	Challenges for deprescription in home care from nurses’ perspectives.
	Sun et al., 2021^34^	Nurses’ educational and learning needs in relation to involvement in deprescription initiatives.
	Tjia et al., 2019^43^	Nurses’ educational needs and involvement of older people and families in medication deprescription.
	Turner et al., 2016^44^	Requirements and factors influencing medication deprescription from healthcare providers’ perspectives.
	Wang et al., 2023^45^	Essential tasks for deprescribing consisting of patient-centredness, and collaboration by the multidisciplinary team involving patients and families.
	Wang et al., 2024^46^	Various challenges to deprescribing and the need for the collaboration of multidisciplinary team along with the inclusion of caregivers.
	Warmoth et al., 2023^47^	Social, contextual, individual factors influencing medication deprescription from healthcare providers, residents and families’ perspectives; family and patient involvement.
Mixed-method design	Azermai et al., 2014^48^	Dose reduction and discontinuation of antipsychotics in 38.1% and 20.5% respectively of the users; an actual reduction and discontinuation in 30.4% and 9.8%, respectively.
	Drewelow et al., 2022^49^	Multidisciplinary team and family involvement for collaborating on deprescription strategies.

CI, confidence interval; HR, hazard ratio; IQR, interquartile range; IRR, incidence rate ratio; PIMs, potentially inappropriate medications; PPIs, proton pump inhibitors; PRN, pro re nata; RR, relative risk.

#### Necessity and benefits of deprescribing

Various medications were mentioned to be the target of deprescription initiatives. They included benzodiazepines,^25,26,31,35,39,42^ antipsychotics,^31,35,41,48,52^ psychotropics such as antidepressants,^27,35,39,54^ gastrointestinal and supplementary medications such as proton-pump inhibitors (PPIs), histamine-2 receptor antagonists, antihistamines, laxatives such as sodium docusate, macrogols and senna, bisphosphonates, acid-regulating drugs, probiotics, multivitamins,^25,32,33,39,52–54^ analgesics^25^ and antihypertensive,^25,28,30,52,53^ As for classification, some studies stated that the target to be general nonessential medications,^32,43^ or prescription and OTC medications.^42^

The benefits of deprescription initiatives were noted to be about improving medication adherence.^42,52^ Also, potential enhancement of quality of life,^41,43,52^ and well-being.^39^ Older people were calmer and displayed more cooperative behaviours and moods and showed improvements in social and physically activity with greater independence in activities of daily living and tailoring individual’s needs on proactively responding to their own needs.^27,37,38^ Moreover, deprescription interventions focusing on prescription and OTC medications addressed concerns for patient safety by mitigating risks associated with a lack of ongoing and essential need for medication use and excessive dosages that might lead to adverse effects such as falls.^24,29,32,37,41–43^

Positive outcomes for the older people also had positive aspects for the nurses, carers and institutions as deprescribing could decrease the time nurses spent on medication administration^52^ and alleviated caregiver administration burden and stress.^43,55^ Deprescribing benefits included reduced healthcare costs,^39,53^ enhancement of family satisfaction as well as better facility and quality indicator scores related to healthcare organizations’ regulatory compliance.^41^

Deprescribing was planned and implemented without creating any negative impact on underlying diseases, alterations in behavioural and psychological symptoms and replacing medications or significantly increasing the use of PRN medications.^30,31,35^ Importantly, any deprescribing needed to occur without causing any adverse outcome including mortality, hospitalizations, falls or restraint use.^25,31,39^

Reducing the routine use or overuse of prescribed medications to manage symptoms and avoiding polypharmacy^26,27,35^ was another identified advantage. Also, deprescribing interventions supported the selection and use of the most suitable medications for managing underlying health conditions while minimizing medication burden and polypharmacy, which consequently simplified and optimized medication regimens.^39,42,53^ In some circumstances, it was believed that behavioural issues could be attributable to underlying health conditions that potentially could be managed without medications or with the use of non-pharmacological approaches such as physical activity and social engagement, redirection, adjustments in routines and family involvement.^41^

#### Multidisciplinary collaboration for deprescribing

The successful deprescription of medications relied on close cooperation among the multidisciplinary pharmaceutical care team. Teamwork and effective communication among members of the team required a multidisciplinary approach and was essential for developing clearer guidelines during the deprescribing process. Teams included primary care physicians, caregivers, home care nurses and pharmacists.^39,45^ Trust between nursing staff and physicians was seen as crucial for effective collaboration during deprescribing.^50^ Nurses found it easier to implement changes when they felt heard by the physician and had a good relationship with them. They preferred a more step-by-step approach for deprescribing with their roles and contributions identified in the process.^50^ Nurses wanted their knowledge about older people to be valued in deprescribing conversations and decisions.^47^

Formal reviews by the pharmaceutical team, given the diverse expertise involved, were seen as ideal opportunities to discuss deprescribing.^37^ Multidisciplinary medication reviews focused on deprescribing regarding drug-related issues and medication use without clinical indication led to decreased mortality and hospitalizations in nursing homes.^29^ Nurses were found to be beneficial for deprescription practices as they assisted physicians to develop understandings of older people’s health conditions and medication responses.^44^ Also, nurses (67.4%) highlighted the need for support and assistance by a clinical pharmacist for deprescribing,^52^ given their specific clinical expertise.^37^ Implementing a deprescribing plan designed by an interprofessional clinical team based on a pharmacist-led medication review showed potential benefits in reducing the doses of PIMs.^25^ In addition, nurses expressed more comfort when a geronto-psychiatrist was involved given the need for specialized training to deprescribe some medications. Access to a mental health team consisting of consultant psychologists or psychiatrists was also noted as a significant benefit.^36,47,50,53^

Communication among nurses, care providers and specialists for deprescription was, nevertheless, often reactive, focusing on discrepancies and errors instead of proactive optimization. This led to difficulties in comprehending and organizing patients’ medication regimens. Unclear and uncoordinated roles among disciplines complicated effective interdisciplinary practice essential for deprescribing.^46^ Individuals such as physicians, nurses, pharmacists and older people prioritized different factors in deprescribing.^44^ For instance, a small proportion of nurses (13.8%) and physicians (12.2%) were willing to discontinue antipsychotics with shared willingness in only 4.2% of cases, indicating differing evaluations of the same person by nurses and physicians.^48^ Physicians were most accepting of deprescribing suggestions by pharmacists for gastrointestinal and anticholinergic drugs (85% and 100% acceptance rates, respectively).^29^

Improvement strategies identified multidisciplinary staff education, fostering a strong care network, collegial mentoring and person-centred care for decision-making to successfully reduce medication use.^28,42^ Establishing processes for deprescribing documentation, monitoring and controlling medication use and structured reviews without increasing the regulatory burdens, could facilitate and streamline deprescription.^36,37,53^ Clear guidelines for addressing patient inquiries within the multidisciplinary pharmaceutical team and a real-time communication platform for sharing documents like medication lists were mentioned as crucial for deprescribing success.^39,45^

#### Nurse role in deprescribing

Nurses played a vital role in the success of deprescription strategies, actively participating in planning, supporting implementation and monitoring outcomes. Their contributions were notably emphasized in these areas. They contributed to care discussions and monitored older people daily, identified side effects that might necessitate dosage adjustments or discontinuation, making their inputs on recommendations and onsite monitoring of deprescribed medications crucial to other healthcare providers’ decision-making.^46,54^ Nurses, as part of daily care, detected symptoms distressing to older people that influenced the prescribing of medications for symptomatic relief and could suggest medication reduction or discontinuation (50.5%) to physicians.^52^ As the primary care provider (hazard ratio (HR): 1.33 (1.13–1.56)), the presence of nurses was correlated with an increased likelihood of bisphosphonate deprescribing.^33^

Nurses identified polypharmacy and informed healthcare providers about clients’ medication information, often resulting from healthcare providers’ incomplete understanding of patients’ medical histories leading to redundant and inappropriate prescriptions.^37,42^

Nurses played a crucial role in educating older people and minimizing their confusions about deprescribing processes.^46^ Given their familiarity with older people and their health conditions and medical records, they were identified as most appropriate to handle communication about deprescription with individuals and their families.^50^

Besides being receptive to a standardized approach for comprehensive medication review during transition,^43^ nurses served as older people’s advocates leveraging their knowledge of individual needs and resources to develop individualized, adaptable and reversible deprescribing process.^47^ Nurses were able to recognize the importance of evaluating older people’s medication needs and support minimizing as many medications as possible through the provision of strategies for PRN medication use for relieving symptoms and completely withdrawing medications.^37,41,46^ In addition, they addressed older people’s concerns and medications’ adverse effects during adjustments and helped mitigate risks associated with deprescribing.^37,39,45^

Pharmacists and physicians emphasized the importance of nurses’ vigilance in monitoring older people’s post-deprescribing to promptly identify any adverse effects, enabling safer medication adjustments and management of potential side effects.^37^ Nurses are eager to deepen their understanding of foundational deprescribing approaches, particularly for medications that pose risks, such as understanding their side effects and interactions with other drugs.^42^ However, a lack of awareness about nurses’ competencies in medication management caused their exclusion from care team discussions further hindering their ability to assist in deprescribing.^46^ On the other hand, lack of confidence and some concerns about the quality of observations from nursing staff due to high staff turnover and varying work shifts raised questions regarding relying on these observations to decide on deprescribing.^37,50^

Nurses mentioned that they needed education and training to support improving their knowledge of how to recognize symptoms/behaviour triggers to respond to residents’ needs, implement nonpharmacological behaviour management strategies and report the result to the team, and build confidence.^37,38^ Training was needed to enhance nurses’ motivation to implement deprescribing in their practice and become more receptive to adopting deprescribing practices within the team.^34^

#### Identified challenges to deprescribing

Nurses have identified several challenges related to knowledge, skill, attitude and resources for implementing deprescription interventions. Lack of sufficient knowledge related to medication management in terms of medications’ risks and benefits, symptoms and their management, discontinuation and deprescription process, identification of patients, caregivers and families’ needs and how they can be engaged were identified challenges in the areas of knowledge and skills.^36,38–40,42,43,47,48,52^

Another challenge was nurses’ concerns about the impact of medication deprescription on older people’s quality of life and well-being, exacerbation of symptoms or conditions and behavioural problems, adverse events and risk of harm.^24,25,39,41,42,48^

Also, nurses’ staffing shortages, high turnover, workload and time constraints for medication review, older people’s education, and identifying issues with deprescribing were stated as other barriers to participation in deprescribing.^36,40,42,46,48^

Complicating planning for medication deprescription were delayed older patient’s encounters after hospital discharge, difficulty reaching care providers and/or delayed home visits.^46^ Additionally, healthcare settings’ regulatory variations in medication administration and increased government oversight affecting providers’ risk-taking abilities,^32,36^ lack of resources, lack of prioritization of deprescribing, inconsistence practices and hesitancy to halt medications prescribed by specialists hindered deprescribing efforts.^24,41,42,46^ The absence of medication review and decision-support tools, such as deprescribing algorithms, guidelines for screening high-risk medications and symptom management, and best practices in medication reconciliation and the use of non-drug therapies posed challenges for implementing deprescription.^34,39,40,42,43,47,51^

Other challenges highlighted were healthcare staff’s overall reluctance to consider patient safety concerns, implement deprescription strategies, older people’s preferences to continue medication therapy and families’ resistance to deprescribing.^24,41,52^

#### Involvement of older people and families in deprescribing

Involving patients, their families and informal caregivers in the deprescription process was identified as crucial for its success, and nurses were in the best position to support it. Their active participation ensured that their preferences, concerns and experiences were considered, fostering a collaborative approach to deprescription efforts. It was found that the older people and their families have the right to access an accurate, comprehensive and updated list of prescriptions, OTC medications and supplements, which facilitated understanding of the need for deprescribing. This included segregating deprescribed medications from actively used ones to prevent errors and ensuring comprehension according to their health literacy levels, along with offering ongoing support. It was also critical to align deprescribing decisions with the patient’s specific goals, needs and preferences and avoid them feeling abandoned or that their medication adherence efforts were wasted.^44,45,49,53,55^ Contrary to this, some healthcare staff were hesitant about involving older people and their families in the deprescribing process, doubting their ability to participate meaningfully and being uncertain about the added value of relatives’ input.^50^ It was stated that nurses had a critical role in initiating deprescribing conversations with older people, families and other healthcare professionals.^34,43^ They could establish care partnerships with older people and families and build trust through direct involvement in care decisions to indicate the inclusion of their preferences for successful deprescribing.^38,43^

It was indicated that all deprescription initiatives should consider developing a network involving older people and informal caregivers along with their education about managing symptoms and the use of non-drug options.^41,42,44^ Education should contain the description of benefits and risks, and older people and their families’ input for shared decision-making given their lack of autonomous confidence based on the complex steps and skills required for deprescribing.^39,47,53,55^

## Discussion

This review aimed to investigate the evolving role of nurses in deprescribing medications in the multidisciplinary pharmaceutical care context of long-term care for older people. The results identified medications being the target and advantages of deprescription, the significance of the close collaboration among the team, nurses’ roles and contributions to support deprescribing, challenges to deprescribing and older people and families’ involvement.

Deprescription interventions targeted both prescribed and OTC medications, categorized as essential or non-essential. The main medication classes highlighted were benzodiazepines, antipsychotics, psychotropics, gastrointestinal, supplements and antihypertensives. According to the international literature, cardiovascular drugs, especially antihypertensives, diuretics and nitrates are often easiest to deprescribe, but psychotropic medications and PPIs require intense deprescription interventions.^56^ Drug-related problems requiring deprescribing or adjusting doses are often psycholeptics, antihypertensives and analgesics.^57^ Stopping or reducing certain drug classes like antihypertensives and cholesterol-lowering drugs has been associated with higher mortality rates.^9^

In our review, deprescribing had benefits for the overall well-being of older people, and the safety of medication management as well as healthcare costs. In general, any intervention aimed at improving medication practices and reducing the exposure of older people to PIM can be associated with a better quality of life among them.^58,59^ Deprescribing interventions for older adults living in the community range from cost-saving to an incremental cost-effectiveness ratio of up to $112,932 per quality-adjusted life-year, exceeding the WHO threshold. Overall, 85% of deprescribing interventions have either saved costs, outperformed usual care, or been considered cost-effective based on a threshold of 1 GDP per capita.^60^

According to our review findings, collaboration and communication among members of the multidisciplinary pharmaceutical team along with coordinated roles among the involved disciplines guided by appropriate guidelines with the recognition of nurses’ roles were found crucial for successful deprescribing. Engaging multidisciplinary teams and reaching consensus on deprescribing decisions are key to successful outcomes.^61^ The participation of each healthcare provider within the team-based medication management, collaborating on education, patient-specific recommendations and close follow-up, has been shown to improve medication safety among community-dwelling older adults.^56,62^ The primary risk factors associated with medication errors during the transition from hospital to community care include inadequate interprofessional communication and the absence of standardized processes for medication reconciliation.^63^ Also, the absence of clearly defined roles causes internal team dynamic conflicts in medication therapy.^64^ Enhancing professional autonomy and providing training can optimize pharmaceutical teamwork.^65^ Also, the presence of nurses is a protective factor against functional decline in nursing home residents^66^ as they significantly influence the quality and safety of healthcare services by adhering to patient safety principles and participating in patient safety initiatives.^67^ They should be recognized for their integral roles in medication management initiatives, as they play a vital role in informing, supporting, representing and engaging all pertinent stakeholders.^68^ Given the need for appropriate support tools for deprescription, computerized clinical decision support systems have been shown to be beneficial and effective with the potential to enhance medication safety in long-term care settings.^69^

Several challenges in the deprescribing process in terms of knowledge and skills, attitude and resources were identified in this review. Deprescription initiatives necessitate costly, intensive interventions and can result in unexpected adverse outcomes that impact individuals.^56^ In general, factors influencing deprescription practices as facilitators and barriers are operational routines, resource availability and staff qualifications, patient-related outcomes such as concerns about the negative effects of discontinuation versus downplaying medication side effects, policies including leadership support, and interprofessional collaboration between staff, beliefs about medication use, staff shortages, resistance from families or residents themselves, proficiency in nonpharmacological approaches, systematic procedures to promote operational efficiency and shared understanding among stakeholders.^61,70–72^

From the clinical perspective, discontinuation can pose significant challenges for patients due to physiological and psychological dependence on medications during the treatment process. It is noted that the discontinuation of psychoactive medications like antipsychotics, benzodiazepines and antidepressants is more complex and presents unique challenges to patients and the healthcare team due to the neuroadaptation they induce. Withdrawal symptoms range from mild discomfort to severe, life-threatening conditions, depending on the medication and duration of use. Stopping psychiatric medications can trigger anxiety, mood changes, insomnia or even seizures. The psychological impact of discontinuation, especially if medications are used to manage chronic health conditions, can exacerbate underlying symptoms, leading to a relapse or worsening of the underlying health condition.^73,74^ Also, patients may face challenges including the resurgence of underlying health conditions, which can be distressing and destabilizing. Therefore, the prescriber should balance the risks of withdrawal symptoms with the potential benefits of deprescribing. Close monitoring by the healthcare team is essential to minimize risks and ensure a smooth transition, emphasizing the need for an individualized approach.^75,76^ Nurses can facilitate smooth medication discontinuation by monitoring patients, managing symptoms, providing reassurance and ensuring prompt communication with the healthcare team and patients.

Based on this review, involving older people and their families ensures that their preferences, concerns and experiences were taken into consideration, promoting a collaborative approach to the process of deprescribing. Older people express a desire to participate in decision-making about their medications and are willing to discontinue one or more medications if recommended by their prescriber, but medication complexity significantly influences their attitudes towards deprescribing (adjusted odds ratio 2.6, 95% CI 1.29–5.29), underscoring the importance of considering medication complexity when making deprescribing decisions.^77^ To avoid challenges, deprescribing outcomes should be clearly defined to facilitate a broader adoption accompanied by adequate follow-up periods and relevant outcome measurement intervals.^78^ The foundation of safe medication management in long-term care, with the involvement of family caregivers, rests on the integration of person-centered values, older people’s needs and the pivotal role of families.^79–81^ Education and support for older people are protective measures that optimize medication therapy, minimize risks associated with medication use and enhance treatment outcomes.^82^ It is essential to develop personalized care plans that prioritize reducing older people’s dependency on medications, empowering family caregivers through role development, education and training, and active involvement in decision-making processes, as well as providing robust support by healthcare professionals.^81^ It is also important to support them in transitioning from discontinued medications to alternative non-drug options, such as physical activity, mind-body practices, acupressure, reflexology and aromatherapy to improve the success of deprescribing interventions by providing effective and holistic alternatives contributing to overall well-being.^78,83,84^

## Limitations and suggestions for future studies

To the best of our knowledge, this is the first systematic review to integrate current international knowledge on the roles and contributions of nurses in deprescribing medications within the multidisciplinary pharmaceutical care context in long-term care for older people. As a limitation, this review excluded studies published before 2014 and those in languages other than English. Future studies should aim to include research published prior to 2014 and in languages other than English to minimize language bias and ensure a more comprehensive analysis of the available evidence. While selection bias was mitigated by including both qualitative and quantitative studies, the challenge of synthesizing data from diverse qualitative and quantitative designs could have influenced the research synthesis.

Expanding nurses’ expertise, competencies and roles within the multidisciplinary deprescription team is a crucial concern requiring further research. Bridging the knowledge gap and detailing nurses’ competencies in this team requires further research to enhance their participation in initiatives aimed at improving medication safety. Clinical guidelines should be developed to support and establish the participation of nurses in deprescription interventions.

## Conclusion

For effective deprescribing, a coordinated, multidisciplinary approach for teamwork and communication among the multidisciplinary pharmaceutical team with the involvement of physicians, nurses, pharmacists, older people, families and caregivers is crucial.

Nurses can play a vital role in supporting the deprescription team by actively participating in care discussions, observing and monitoring older people in long-term care settings daily, and providing valuable insights into medication management. They can communicate with and educate older people and their families and caregivers, alleviate their confusion, and identify deprescription’s side effects. Nurses can also address polypharmacy by highlighting concerns and providing critical medication information to physicians and pharmacists. By sharing their detailed knowledge of older people’s medications and health conditions, developing personalized deprescription plans and implementing PRN strategies and non-pharmacologic methods, they can minimize unnecessary medication use and vigilantly monitor them during medication adjustments. Therefore, nurses’ role and participation should be valued and should be empowered through ongoing education and training regarding deprescribing to foster a robust medication management team.

There is also a need to improve the attitudes of healthcare staff, older people and their families about the necessity of deprescribing and its risks and benefits. Also, a deprescribing guideline that clearly identifies the roles and contributions of all involved disciplines and how they communicate and collaborate, considers healthcare organizations’ regulatory aspects and provides required facilities and resources should be developed.

Active participation of older people and their families in the deprescription process should be encouraged to ensure their preferences, concerns and experiences are considered. A collaborative approach to decision-making should be fostered with them to enhance the success of deprescribing in long-term care settings and improve the overall safety of medication management for older people.

## Supplemental Material

sj-docx-1-taw-10.1177_20420986241289205 – Supplemental material for An integrative systematic review of nurses’ involvement in medication deprescription in long-term healthcare settings for older people Supplemental material, sj-docx-1-taw-10.1177_20420986241289205 for An integrative systematic review of nurses’ involvement in medication deprescription in long-term healthcare settings for older people by Mojtaba Vaismoradi, Abbas Mardani, Manuel Lillo Crespo, Patricia A. Logan and Natalia Sak-Dankosky in Therapeutic Advances in Drug Safety

sj-docx-2-taw-10.1177_20420986241289205 – Supplemental material for An integrative systematic review of nurses’ involvement in medication deprescription in long-term healthcare settings for older people Supplemental material, sj-docx-2-taw-10.1177_20420986241289205 for An integrative systematic review of nurses’ involvement in medication deprescription in long-term healthcare settings for older people by Mojtaba Vaismoradi, Abbas Mardani, Manuel Lillo Crespo, Patricia A. Logan and Natalia Sak-Dankosky in Therapeutic Advances in Drug Safety

sj-docx-3-taw-10.1177_20420986241289205 – Supplemental material for An integrative systematic review of nurses’ involvement in medication deprescription in long-term healthcare settings for older people Supplemental material, sj-docx-3-taw-10.1177_20420986241289205 for An integrative systematic review of nurses’ involvement in medication deprescription in long-term healthcare settings for older people by Mojtaba Vaismoradi, Abbas Mardani, Manuel Lillo Crespo, Patricia A. Logan and Natalia Sak-Dankosky in Therapeutic Advances in Drug Safety

sj-docx-4-taw-10.1177_20420986241289205 – Supplemental material for An integrative systematic review of nurses’ involvement in medication deprescription in long-term healthcare settings for older people Supplemental material, sj-docx-4-taw-10.1177_20420986241289205 for An integrative systematic review of nurses’ involvement in medication deprescription in long-term healthcare settings for older people by Mojtaba Vaismoradi, Abbas Mardani, Manuel Lillo Crespo, Patricia A. Logan and Natalia Sak-Dankosky in Therapeutic Advances in Drug Safety

## Declarations

**Ethics approval and consent to participate:** Not applicable.

**Consent for publication:** Not applicable.

**Availability of data and materials:** The data that support the findings of this review are available upon a reasonable request from the corresponding author.

## References

[1] World Health Organization. Ageing and health, https://www.who.int/news-room/fact-sheets/detail/ageing-and-health (2022, accessed 25 June 2024).

[2] Martinez-Lacoba R Pardo-Garcia I Escribano-Sotos F. Aging, dependence, and long-term care: a systematic review of employment creation. Inquiry 2021; 58: 469580211062426. 10.1177/00469580211062426 PMC8695749 34913376

[3] OECD. Long-term care for older people: the OECD Health Project, https://www.oecd.org/en/topics/sub-issues/ageing-and-long-term-care.html (2005, accessed 16 August 2024).

[4] Facchinetti G D’Angelo D Piredda M , et al. Continuity of care interventions for preventing hospital readmission of older people with chronic diseases: a meta-analysis. Int J Nurs Stud 2020; 101: 103396. 31698168 10.1016/j.ijnurstu.2019.103396

[5] Tomlinson J Cheong VL Fylan B , et al. Successful care transitions for older people: a systematic review and meta-analysis of the effects of interventions that support medication continuity. Age Ageing 2020; 49: 558–569. 32043116 10.1093/ageing/afaa002 PMC7331096

[6] Bick I Dowding D. Hospitalization risk factors of older cohorts of home health care patients: a systematic review. Home Health Care Serv Q 2019; 38: 111–152. 31100045 10.1080/01621424.2019.1616026

[7] Jokanovic N Tan EC Dooley MJ , et al. Prevalence and factors associated with polypharmacy in long-term care facilities: a systematic review. J Am Med Dir Assoc 2015; 16: 535.e1–535.e12. 10.1016/j.jamda.2015.03.003 25869992

[8] Manirajan P Sivanandy P. Drug utilisation review among geriatric patients with noncommunicable diseases in a primary care setting in Malaysia. Healthcare (Basel) 2023; 11: 1665. 10.3390/healthcare11121665 PMC10298635 37372782

[9] Russell P Hewage U McDonald C , et al. Prospective cohort study of nonspecific deprescribing in older medical inpatients being discharged to a nursing home. Ther Adv Drug Saf 2021; 12: 20420986211052344. 10.1177/20420986211052344 PMC8543714 34707803

[10] Glans M Kragh Ekstam A Jakobsson U , et al. Risk factors for hospital readmission in older adults within 30 days of discharge – a comparative retrospective study. BMC Geriatr 2020; 20: 467. 33176721 10.1186/s12877-020-01867-3 PMC7659222

[11] Linkens A Milosevic V van der Kuy PHM , et al. Medication-related hospital admissions and readmissions in older patients: an overview of literature. Int J Clin Pharm 2020; 42: 1243–1251. 32472324 10.1007/s11096-020-01040-1 PMC7522062

[12] Achterhof AB Rozsnyai Z Reeve E , et al. Potentially inappropriate medication and attitudes of older adults towards deprescribing. PLoS One 2020; 15: e0240463. 10.1371/journal.pone.0240463 PMC7588126 33104695

[13] Hammami S Zarrouk A Piron C , et al. Prevalence and factors associated with frailty in hospitalized older patients. BMC Geriatr 2020; 20: 144. 32306905 10.1186/s12877-020-01545-4 PMC7168944

[14] Wang KN Bell JS Chen EYH , et al. Medications and prescribing patterns as factors associated with hospitalizations from long-term care facilities: a systematic review. Drugs Aging 2018; 35: 423–457. 29582403 10.1007/s40266-018-0537-3

[15] Wang J Shen JY Conwell Y , et al. Antipsychotic use among older patients with dementia receiving home health care services: prevalence, predictors, and outcomes. J Am Geriatr Soc 2023; 71: 3768–3779. 37671461 10.1111/jgs.18555 PMC10841208

[16] Wu H Kouladjian O’Donnell L Fujita K , et al. Deprescribing in the older patient: a narrative review of challenges and solutions. Int J Gen Med 2021; 14: 3793–3807. 34335046 10.2147/IJGM.S253177 PMC8317936

[17] Edey R Edwards N Von Sychowski J , et al. Impact of deprescribing rounds on discharge prescriptions: an interventional trial. Int J Clin Pharm 2019; 41: 159–166. 30478496 10.1007/s11096-018-0753-2

[18] Kua CH Mak VSL Huey Lee SW. Health outcomes of deprescribing interventions among older residents in nursing homes: a systematic review and meta-analysis. J Am Med Dir Assoc 2019; 20: 362.e11–372.e11. 10.1016/j.jamda.2018.10.026 30581126

[19] Vogelsmeier A Anderson RA Anbari A , et al. A qualitative study describing nursing home nurses sensemaking to detect medication order discrepancies. BMC Health Serv Res 2017; 17: 531. 28778158 10.1186/s12913-017-2495-6 PMC5545015

[20] Cool C Cestac P McCambridge C , et al. Reducing potentially inappropriate drug prescribing in nursing home residents: effectiveness of a geriatric intervention. Br J Clin Pharmacol 2018; 84: 1598–1610. 29607568 10.1111/bcp.13598 PMC6005629

[21] Whittemore R Knafl K. The integrative review: updated methodology. J Adv Nurs 2005; 52: 546–553. 16268861 10.1111/j.1365-2648.2005.03621.x

[22] Hong QN PP Fàbregues S Bartlett G , et al. Mixed Methods Appraisal Tool (MMAT), version 2018, http://mixedmethodsappraisaltoolpublic.pbworks.com/w/file/fetch/127916259/MMAT_2018_criteria-manual_2018-08-01_ENG.pdf (2018, accessed 15 September 2024).

[23] Risk of Bias.info. Risk of bias tools for use in systematic reviews, Risk of bias tools, https://www.riskofbias.info/ (2024, accessed 25 June 2024).

[24] Balsom C Pittman N King R , et al. Impact of a pharmacist-administered deprescribing intervention on nursing home residents: a randomized controlled trial. Int J Clin Pharm 2020; 42: 1153–1167. 32494991 10.1007/s11096-020-01073-6

[25] Cateau D Ballabeni P Niquille A. Effects of an interprofessional deprescribing intervention in Swiss nursing homes: the Individual Deprescribing Intervention (IDeI) randomised controlled trial. BMC Geriatr 2021; 21: 655. 34798826 10.1186/s12877-021-02465-7 PMC8603597

[26] Evrard P Henrard S Foulon V , et al. Benzodiazepine use and deprescribing in Belgian nursing homes: results from the COME-ON study. J Am Geriatr Soc 2020; 68: 2768–2777. 32786002 10.1111/jgs.16751

[27] Gedde MH Husebo BS Mannseth J , et al. Less is more: the impact of deprescribing psychotropic drugs on behavioral and psychological symptoms and daily functioning in nursing home patients. Results from the cluster-randomized controlled COSMOS Trial. Am J Geriatr Psychiatry 2021; 29: 304–315. 32753339 10.1016/j.jagp.2020.07.004

[28] Gulla C Flo E Kjome RL , et al. Deprescribing antihypertensive treatment in nursing home patients and the effect on blood pressure. J Geriatr Cardiol 2018; 15: 275–283. 29915617 10.11909/j.issn.1671-5411.2018.04.011 PMC5997621

[29] Kua CH Yeo CYY Tan PC , et al. Association of deprescribing with reduction in mortality and hospitalization: a pragmatic stepped-wedge cluster-randomized controlled trial. J Am Med Dir Assoc 2021; 22: 82.e3–89.e3. 10.1016/j.jamda.2020.03.012 32423694

[30] Sheppard JP Burt J Lown M , et al. Effect of antihypertensive medication reduction vs usual care on short-term blood pressure control in patients with hypertension aged 80 years and older: the OPTIMISE randomized clinical trial. JAMA 2020; 323: 2039–2051. 32453368 10.1001/jama.2020.4871 PMC7251449

[31] Brodaty H Aerts L Harrison F , et al. Antipsychotic deprescription for older adults in long-term care: the HALT study. J Am Med Dir Assoc 2018; 19: 592.e7–600.e7. 10.1016/j.jamda.2018.05.002 29941156

[32] McConeghy KW Cinque M White EM , et al. Lessons for deprescribing from a nonessential medication hold policy in US nursing homes. J Am Geriatr Soc 2022; 70: 429–438. 34695233 10.1111/jgs.17512 PMC8821115

[33] Niznik JD Aspinall SL Hanson LC , et al. Patterns of oral bisphosphonate deprescribing in older nursing home residents with dementia. Osteoporos Int 2022; 33: 379–390. 34480586 10.1007/s00198-021-06141-9 PMC8813888

[34] Sun W Tahsin F Abbass Dick J , et al. Educating homecare nurses about deprescribing of medications to manage polypharmacy for older adults. West J Nurs Res 2021; 43: 193945920982599. 10.1177/0193945920982599 PMC8495304 33435859

[35] Westbury JL Gee P Ling T , et al. RedUSe: reducing antipsychotic and benzodiazepine prescribing in residential aged care facilities. Med J Aust 2018; 208: 398–403. 29747564 10.5694/mja17.00857

[36] Abrahamson K Davila H Kirk L , et al. Can a nursing home psychotropic reduction project be successfully implemented in assisted living? J Appl Gerontol 2021; 40: 1071–1079. 32772612 10.1177/0733464820948328

[37] Birt L Wright DJ Blacklock J , et al. Enhancing deprescribing: a qualitative understanding of the complexities of pharmacist-led deprescribing in care homes. Health Soc Care Community 2022; 30: e6521–e6531. 10.1111/hsc.14099 PMC10100492 36336895

[38] Chenoweth L Jessop T Harrison F , et al. Critical contextual elements in facilitating and achieving success with a person-centred care intervention to support antipsychotic deprescribing for older people in long-term care. Biomed Res Int 2018; 2018: 7148515. 30069476 10.1155/2018/7148515 PMC6057399

[39] Kua CH Mak VS Lee SWH . Perspectives of health professionals towards deprescribing practice in Asian nursing homes: a qualitative interview study. BMJ Open 2019; 9: e030106. 10.1136/bmjopen-2019-030106 PMC6797246 31604786

[40] Palagyi A Keay L Harper J , et al. Barricades and brickwalls – a qualitative study exploring perceptions of medication use and deprescribing in long-term care. BMC Geriatr 2016; 16: 15. 26767619 10.1186/s12877-016-0181-x PMC4714480

[41] Simmons SF Bonnett KR Hollingsworth E , et al. Reducing antipsychotic medication use in nursing homes: a qualitative study of nursing staff perceptions. Gerontologist 2018; 58: e239–e250. 10.1093/geront/gnx083 28575301

[42] Sun W Tahsin F Barakat-Haddad C , et al. Exploration of home care nurse’s experiences in deprescribing of medications: a qualitative descriptive study. BMJ Open 2019; 9: e025606. 10.1136/bmjopen-2018-025606 PMC6538031 31129579

[43] Tjia J DeSanto-Madeya S Mazor KM , et al. Nurses’ perspectives on family caregiver medication management support and deprescribing. J Hosp Palliat Nurs 2019; 21: 312–318. 31033645 10.1097/NJH.0000000000000574 PMC7561076

[44] Turner JP Edwards S Stanners M , et al. What factors are important for deprescribing in Australian long-term care facilities? Perspectives of residents and health professionals. BMJ Open 2016; 6: e009781. 10.1136/bmjopen-2015-009781 PMC4800122 26966056

[45] Wang J Shen JY Yu F , et al. How to deprescribe potentially inappropriate medications during the hospital-to-home transition: stakeholder perspectives on essential tasks. Clin Ther 2023; 45: 947–956. 37640614 10.1016/j.clinthera.2023.07.023 PMC10841554

[46] Wang J Shen JY Yu F , et al. Challenges in deprescribing among older adults in post-acute care transitions to home. J Am Med Dir Assoc 2024; 25: 138.e6–145.e6. 10.1016/j.jamda.2023.09.021 PMC10843747 37913819

[47] Warmoth K Rees J Day J , et al. Determinants of implementing deprescribing for older adults in English care homes: a qualitative interview study. BMJ Open 2023; 13: e081305. 10.1136/bmjopen-2023-081305 PMC10668129 37996237

[48] Azermai M Vander Stichele RR Van Bortel LM , et al. Barriers to antipsychotic discontinuation in nursing homes: an exploratory study. Aging Ment Health 2014; 18: 346–353. 24015865 10.1080/13607863.2013.832732

[49] Drewelow E Ritzke M Altiner A , et al. Development of a shared decision-making intervention to improve drug safety and to reduce polypharmacy in frail elderly patients living at home. PEC Innov 2022; 1: 100032. 37213749 10.1016/j.pecinn.2022.100032 PMC10194292

[50] Hølmkjær P Vermehren C Holm A , et al. Tailoring a complex intervention to reduce antidepressants in institutionalized older persons with dementia. BMC Health Serv Res 2022; 22: 1582. 36572903 10.1186/s12913-022-08961-9 PMC9791154

[51] Perri GA Bortolussi-Courval É Brinton CD , et al. MedSafer to support deprescribing for residents of long-term care: a mixed-methods study. Can Geriatr J 2022; 25: 175–182. 35747414 10.5770/cgj.25.545 PMC9156423

[52] Ailabouni N Tordoff J Mangin D , et al. Do residents need all their medications? A cross-sectional survey of RNs’ views on deprescribing and the role of clinical pharmacists. J Gerontol Nurs 2017; 43: 13–20. 10.3928/00989134-20170914-05 28945268

[53] Baqir W Hughes J Jones T , et al. Impact of medication review, within a shared decision-making framework, on deprescribing in people living in care homes. Eur J Hosp Pharm 2017; 24: 30–33. 31156894 10.1136/ejhpharm-2016-000900 PMC6451483

[54] Pruskowski J Handler SM. The DE-PHARM project: a pharmacist-driven deprescribing initiative in a nursing facility. Consult Pharm 2017; 32: 468–478. 29029668 10.4140/TCP.n.2017.468

[55] Pruskowski J Sakely H Handler S. Development of a required longitudinal residency experience focused on deprescribing. Am J Health Syst Pharm 2019; 76: 236–241. 31415680 10.1093/ajhp/zxy029

[56] Dills H Shah K Messinger-Rapport B , et al. Deprescribing medications for chronic diseases management in primary care settings: a systematic review of randomized controlled trials. J Am Med Dir Assoc 2018; 19: 923.e2–935.e2. 10.1016/j.jamda.2018.06.021 30108032

[57] Gangoso Fermoso A Herrero Domínguez-Berrueta MC Pipaon MRP , et al. [Multidisciplinar revision of treatment in nursing home patients in COVID-19 context]. J Healthc Qual Res 2022; 37: 34–43. 34417158 10.1016/j.jhqr.2021.07.002 PMC8292030

[58] Clark CM Guan J Patel AR , et al. Association between potentially inappropriate medications prescription and health-related quality of life among US older adults. J Am Geriatr Soc 2024; 72(9): 2807–2815. 38725422 10.1111/jgs.18957 PMC11904790

[59] Khezrian M McNeil CJ Murray AD , et al. An overview of prevalence, determinants and health outcomes of polypharmacy. Ther Adv Drug Saf 2020; 11: 2042098620933741. 10.1177/2042098620933741 PMC7294476 32587680

[60] Romano S Figueira D Teixeira I , et al. Deprescribing interventions among community-dwelling older adults: a systematic review of economic evaluations. Pharmacoeconomics 2022; 40: 269–295. 34913143 10.1007/s40273-021-01120-8

[61] Paque K Vander Stichele R Elseviers M , et al. Barriers and enablers to deprescribing in people with a life-limiting disease: a systematic review. Palliat Med 2019; 33: 37–48. 30229704 10.1177/0269216318801124

[62] Abu Fadaleh SM Charrois TL Makhinova T , et al. The effect of home medication review in community-dwelling older adults: a systematic review. J Public Health 2022; 30: 1857–1872.

[63] Dionisi S Di Simone E Liquori G , et al. Medication errors’ causes analysis in home care setting: a systematic review. Public Health Nurs 2022; 39: 876–897. 34967458 10.1111/phn.13037

[64] Ferreri SP Hughes TD Snyder ME. Medication therapy management: current challenges. Integr Pharm Res Pract 2020; 9: 71–81. 32309200 10.2147/IPRP.S179628 PMC7136570

[65] Garland CT Guénette L Kröger E , et al. A new care model reduces polypharmacy and potentially inappropriate medications in long-term care. J Am Med Dir Assoc 2021; 22: 141–147. 33221164 10.1016/j.jamda.2020.09.039

[66] Moreno-Martin P Jerez-Roig J Rierola-Fochs S , et al. Incidence and predictive factors of functional decline in older people living in nursing homes: a systematic review. J Am Med Dir Assoc 2022; 23: 1815.e9–1825.e9. 10.1016/j.jamda.2022.05.001 35679882

[67] Vaismoradi M Tella S Logan PA , et al. Nurses’ adherence to patient safety principles: a systematic review. Int J Environ Res Public Health 2020; 17: 2028. 32204403 10.3390/ijerph17062028 PMC7142993

[68] Huisman BAA Geijteman ECT Dees MK , et al. Role of nurses in medication management at the end of life: a qualitative interview study. BMC Palliat Care 2020; 19: 68. 32404166 10.1186/s12904-020-00574-5 PMC7222510

[69] Marasinghe KM. Computerised clinical decision support systems to improve medication safety in long-term care homes: a systematic review. BMJ Open 2015; 5: e006539. 10.1136/bmjopen-2014-006539 PMC4431065 25967986

[70] Kvarnström K Westerholm A Airaksinen M , et al. Factors contributing to medication adherence in patients with a chronic condition: a scoping review of qualitative research. Pharmaceutics 2021; 13(7): 1100. 34371791 10.3390/pharmaceutics13071100 PMC8309154

[71] Moth AE Hølmkjær P Holm A , et al. What makes deprescription of psychotropic drugs in nursing home residents with dementia so challenging? A qualitative systematic review of barriers and facilitators. Drugs Aging 2021; 38: 671–685. 34231182 10.1007/s40266-021-00875-1 PMC8342345

[72] Raza A Piekarz H Jawad S , et al. A systematic review of quantitative studies exploring staff views on antipsychotic use in residents with dementia in care homes. Int J Clin Pharm 2023; 45: 1050–1061. 37773304 10.1007/s11096-023-01645-2 PMC10600045

[73] Fava GA Cosci F. Understanding and managing withdrawal syndromes after discontinuation of antidepressant drugs. J Clin Psychiatry 2019; 80: 19com12794. 10.4088/JCP.19com12794 31774947

[74] Reeve E Thompson W Farrell B. Deprescribing: a narrative review of the evidence and practical recommendations for recognizing opportunities and taking action. Eur J Intern Med 2017; 38: 3–11. 28063660 10.1016/j.ejim.2016.12.021

[75] Jansen J Naganathan V Carter SM , et al. Too much medicine in older people? Deprescribing through shared decision making. BMJ 2016; 353: i2893. 10.1136/bmj.i2893 27260319

[76] Cosci F Chouinard G. Acute and persistent withdrawal syndromes following discontinuation of psychotropic medications. Psychother Psychosom 2020; 89: 283–306. 32259826 10.1159/000506868

[77] Mohammed MA Harrison J Milosavljevic A , et al. Attitude towards deprescribing and its association with frailty and complexity of medication regimen: a survey of older inpatients in a district health board in New Zealand. BMC Geriatr 2023; 23: 166. 36959598 10.1186/s12877-023-03878-2 PMC10035261

[78] Nizet P Evin A Brociero E , et al. Outcomes in deprescribing implementation trials and compliance with expert recommendations: a systematic review. BMC Geriatr 2023; 23: 428. 37438697 10.1186/s12877-023-04155-y PMC10337166

[79] Anker-Hansen C Skovdahl K McCormack B , et al. The third person in the room: the needs of care partners of older people in home care services – a systematic review from a person-centred perspective. J Clin Nurs 2018; 27: e1309–e1326. 10.1111/jocn.14205 29194850

[80] Boland L Légaré F Perez MM , et al. Impact of home care versus alternative locations of care on elder health outcomes: an overview of systematic reviews. BMC Geriatr 2017; 17: 20. 28088166 10.1186/s12877-016-0395-y PMC5237488

[81] Vaismoradi M Jamshed S Lorenzl S , et al. PRN medicines management for older people with long-term mental health disorders in home care. Risk Manag Healthc Policy 2021; 14: 2841–2849. 34262371 10.2147/RMHP.S316744 PMC8274703

[82] Rosengren K Szemberg C. Ensuring safe medication assessment for older adults: a pilot study. Home Health Care Manage Pract. Epub ahead of print 6 June 2024. DOI:10.1177/10848223241257498.

[83] Peng Y Liu Y Guo Z , et al. Doll therapy for improving behavior, psychology and cognition among older nursing home residents with dementia: a systematic review and meta-analysis. Geriatr Nurs 2024; 55: 119–129. 37980780 10.1016/j.gerinurse.2023.10.025

[84] Shang B Yin H Jia Y , et al. Nonpharmacological interventions to improve sleep in nursing home residents: a systematic review. Geriatr Nurs 2019; 40: 405–416. 30795838 10.1016/j.gerinurse.2019.01.001

