# Supplementary material for: An integrative systematic review of nurses’ involvement in medication deprescription in long-term healthcare settings for older people
Source: Ther Adv Drug Saf. 2024 Oct 16;15:20420986241289205. doi: 10.1177/20420986241289205 (PMC11487518; doi:10.1177/20420986241289205)
Supplement: sj-docx-2-taw-10.1177_20420986241289205 – Supplemental material for An integrative systematic review of nurses’ involvement in medication deprescription in long-term healthcare settings for older people [file sj-docx-2-taw-10.1177_20420986241289205.docx]

**Supplementary file 2.** Key words used in the search process

medicat* OR drug* OR medicines OR “medicines management” OR “medication management” AND old* OR elder* OR geriatr* OR aged* OR senior*

AND “nurs*” OR “nurse-led” OR “nurse-driven” OR “nurse-managed” OR “nursing-directed” OR “nursing-led care” OR “nurse-led intervention” OR “nurse-led program” OR “nurse-led initiative” OR “nurse-led decision-making”

AND “home care” OR home OR “home health care” OR “in-home care” OR “home-based care” OR “aging in place” OR “home health nursing” OR “home care services” OR “home care nursing” OR “home health aides” OR “home care for seniors” OR “elderly care at home” OR “home care assistance” OR “home health care agencies” OR “home care for disabled” OR “palliative home care” OR “home care for chronic illness” OR “home care for older patients” OR “home care support” OR “respite home care” OR “residential facility” OR “skilled nursing facility” OR “nursing home” OR “home nursing”

AND “deprescribing” OR “deprescription” OR “medication reduction” OR “medication optimization” OR “rational prescribing” OR “drug withdrawal” OR “dose reduction” OR “de-escalation of medication” OR “drug discontinuation” OR “medication discontinuation” OR “safe deprescribing”
