# Supplementary material for: An integrative systematic review of nurses’ involvement in medication deprescription in long-term healthcare settings for older people
Source: Ther Adv Drug Saf. 2024 Oct 16;15:20420986241289205. doi: 10.1177/20420986241289205 (PMC11487518; doi:10.1177/20420986241289205)
Supplement: sj-docx-3-taw-10.1177_20420986241289205 – Supplemental material for An integrative systematic review of nurses’ involvement in medication deprescription in long-term healthcare settings for older people [file sj-docx-3-taw-10.1177_20420986241289205.docx]

**Supplementary file 3**. The result of the search process

| **Databases from 2014-2024 (last decade)** | **Total in each database** | **Results after title and abstract reading** | **Results after full-text reading and quality appraisal** |
| --- | --- | --- | --- |
| PubMed (including Medline) | 1209 | 20 | 8 |
| Scopus | 348 | 17 | 9 |
| Cinahl | 2816 | 11 | 4 |
| Embase | 288 | 1 | 0 |
| ProQuest | 211 | 14 | 10 |
| Manual search/backtracking references | --- | 17 | 1 |
| **Total of databases** | 4872 | 63 | 32 |
| **Duplications** | 635 | - | --- |
