# Supplementary material for: An integrative systematic review of nurses’ involvement in medication deprescription in long-term healthcare settings for older people
Source: Ther Adv Drug Saf. 2024 Oct 16;15:20420986241289205. doi: 10.1177/20420986241289205 (PMC11487518; doi:10.1177/20420986241289205)

**Supplementary file 4.**

Quality appraisal of quantitative randomized controlled trials using the Mixed Methods Appraisal Tool (MMAT), version 2018

| Authors, Year | Is randomization appropriately performed? | Are the groups comparable at baseline? | Are there complete outcome data? | Are outcome assessors blinded to the intervention provided? | Did the participants adhere to the assigned intervention? |
| --- | --- | --- | --- | --- | --- |
| Balsom et al., 2020 | Yes | Yes | Yes | Not evident | Yes |
| Cateau et al., 2021 | Yes | Yes | Yes | No | Yes |
| Evrard et al., 2020 | Yes | Yes | No | Yes | Yes |
| Gedde et al., 2021 | Yes | Yes | No | Not evident | Yes |
| Gulla et al., 2018 | Yes | Yes | Yes | No | Not evident |
| Kua et al., 2021 | Yes | Yes | Yes | No | Yes |
| Sheppard et al., 2020 | Yes | Yes | Yes | No | Yes |

Quality appraisal of quantitative non-randomized studies using the Mixed Methods Appraisal Tool (MMAT), version 2018

| Authors, Year | Are the participants representative of the target population? | Are measurements appropriate regarding both the outcome and intervention (or exposure)? | Are there complete outcome data? | Are the confounders accounted for in the design and analysis? | During the study period, is the intervention administered (or exposure occurred) as intended? |
| --- | --- | --- | --- | --- | --- |
| Brodaty et al., 2018 | No | Yes | No | Yes | Not evident |
| McConeghy et al., 2022 | Yes | Yes | Yes | Yes | Yes |
| Niznik et al., 2022 | No | Yes | Yes | Yes | Yes |
| Sun et al., 2021 | Yes | Yes | Yes | No | Yes |
| Westbury et al., 2018 | Yes | Yes | Yes | Yes | Yes |

Quality appraisal of qualitative studies using the Mixed Methods Appraisal Tool (MMAT), version 2018

| Authors, Year | Is the qualitative approach appropriate to answer the research question? | Are the qualitative data collection methods adequate to address the research question? | Are the findings adequately derived from the data? | Is the interpretation of results sufficiently substantiated by data? | Is there coherence between qualitative data sources, collection, analysis and interpretation? |
| --- | --- | --- | --- | --- | --- |
| Abrahamson et al., 2021 | Yes | Yes | Yes | Yes | Yes |
| Birt et al., 2022 | Yes | Yes | Yes | Yes | Yes |
| Chenoweth et al., 2018 | Yes | Yes | Yes | No | Yes |
| Kua et al., 2019 | Yes | Yes | Yes | Yes | Yes |
| Palagy et al., 2016 | Yes | Yes | Yes | Yes | Yes |
| Simmons, et al., 2018 | Yes | Yes | Yes | No | Yes |
| Sun et al., 2019 | Yes | Yes | No | No | Yes |
| Tjia et al., 2019 | Yes | No | Yes | Yes | Yes |
| Turner et al., 2016 | Yes | Yes | Yes | Yes | Yes |
| Wang et al., 2024 | Yes | Yes | Yes | Yes | Yes |
| Wang et al., 2023 | Yes | Yes | Yes | Yes | Yes |
| Warmoth et al., 2023 | Yes | No | Yes | Yes | Yes |

Quality appraisal of mixed method studies using the Mixed Methods Appraisal Tool (MMAT), version 2018

| Authors, Year | Is there an adequate rationale for using a mixed methods design to address the research question? | Are the different components of the study effectively integrated to answer the research question? | Are the outputs of the integration of qualitative and quantitative components adequately interpreted? | Are divergences and inconsistencies between quantitative and qualitative results adequately addressed? | Do the different components of the study adhere to the quality criteria of each tradition of the methods involved? |
| --- | --- | --- | --- | --- | --- |
| Azermai, et al., 2014 | Yes | Yes | Yes | Yes | Yes |
| Drewelow et al., 2022 | Yes | Yes | Yes | Yes | Yes |
| Hølmkjær et al., 2022 | Yes | Yes | Yes | Yes | Yes |
| Perri et al., 2022 | Yes | Yes | Yes | Yes | Yes |

Quality appraisal of quantitative descriptive studies using the Mixed Methods Appraisal Tool (MMAT), version 2018

| Authors, Year | Is the sampling strategy relevant to address the research question? | Is the sample representative of the target population? | Are the measurements appropriate? | Is the risk of nonresponse bias low? | Is the statistical analysis appropriate to answer the research question? |
| --- | --- | --- | --- | --- | --- |
| Ailabouni, et al., 2017 | Yes | Yes | Yes | No | Yes |
| Baqir et al., 2017 | Yes | No | Yes | Not evident | No |
| Pruskowsk and Handler, 2017 | Yes | No | Yes | No | No |
| Pruskowski et al., 2019 | Yes | No | Yes | No | No |

Visualization of the risk of bias assessment in randomized clinical trial studies


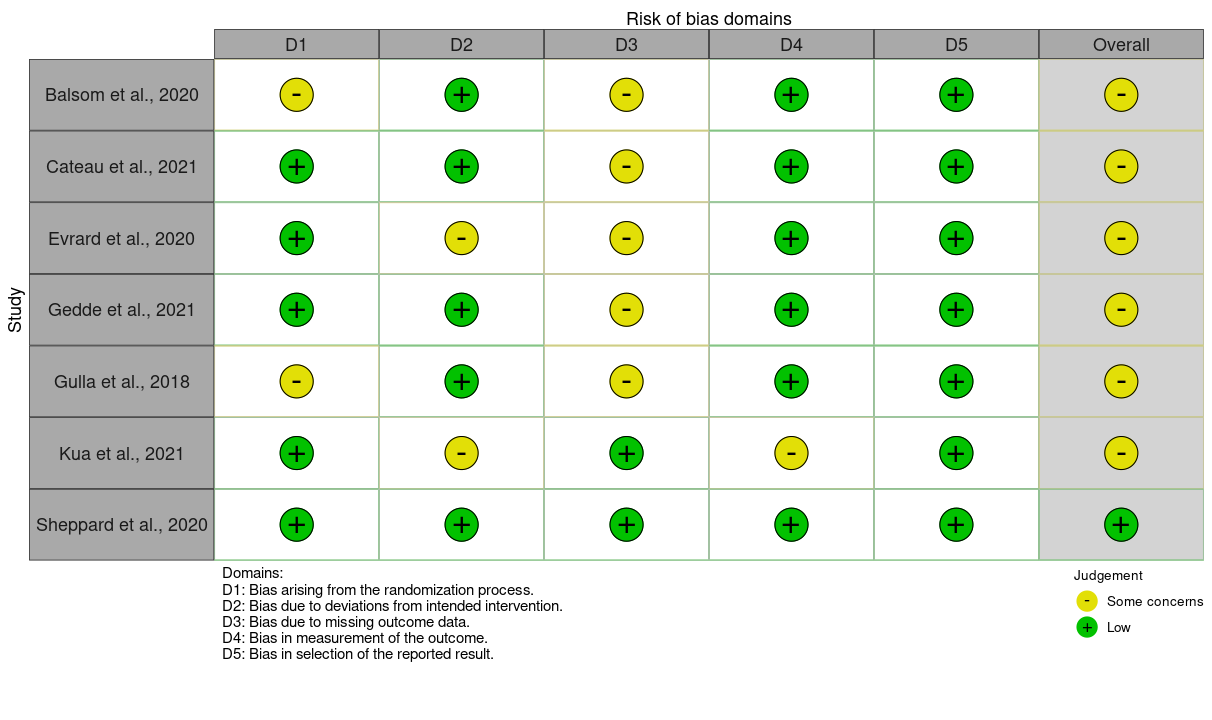

Supplement: sj-docx-4-taw-10.1177_20420986241289205 – Supplemental material for An integrative systematic review of nurses’ involvement in medication deprescription in long-term healthcare settings for older people [file sj-docx-4-taw-10.1177_20420986241289205.docx]
